# A Comparative Study of Sodium Houttuyfonate and 2-Undecanone for Their *in Vitro* and *in Vivo* Anti-Inflammatory Activities and Stabilities

**DOI:** 10.3390/ijms151222978

**Published:** 2014-12-11

**Authors:** Jing Chen, Wenqing Wang, Chunyang Shi, Jianguo Fang

**Affiliations:** Department of Pharmacy, Tongji Hospital, Tongji Medical College, Huazhong University of Science and Technology, Wuhan 430030, China; E-Mails: cj8004@163.com (J.C.); wwq3560@sina.com (W.W.); scy730512@sina.com (C.S.)

**Keywords:** sodium houttuyfonate, 2-undecanone, anti-inflammatory activity, stability, bioavailability, gas chromatography-mass spectrometry (GC-MS)

## Abstract

*Houttuynia cordata* Thunb. (*H. cordata*) is an anti-inflammatory herbal drug that is clinically used in Asia. The essential oil obtained from *H. cordata* is known to contain 2-undecanone (2-methyl nonyl ketone). In addition, sodium houttuyfonate is a compound that can be derived from *H. cordata* and has important clinical uses as an anti-inflammatory agent. Sodium houttuyfonate can be converted to decanoyl acetaldehyde (houttuynin) and then to 2-undecanone. Therefore, the experiments described here explore the comparative anti-inflammatory activities of these compounds. Sodium houttuyfonate showed more potent anti-inflammatory activities than that of 2-undecanone at the same dosage, both *in vitro* and *in vivo*, although both compounds significantly inhibited the production of tumor necrosis factor-α (TNF-α), interleukin-1β (IL-1β) and the expression of toll-like receptor 4 (TLR4), but increased the secretion of interleukin-10 (IL-10) in lipopolysaccharide (LPS)-stimulated RAW264.7 cells. In addition, both compounds showed dose-dependent inhibitory effects on xylene-induced mouse ear edema. In a previous study, we found sodium houttuyfonate to be transformed to 2-undecanone during steam distillation (SD). Optimum therapeutic effects are related to the stability and pharmacological activity of the drugs. Consequently, we studied the stability of sodium houttuyfonate under a simulated gastrointestinal environment with the main influencing factors being solvent, temperature and pH effects. For the first time, sodium houttuyfonate and 2-undecanone were detected simultaneously in the mouse serum and the gastrointestinal tissue after oral administration. Sodium houttuyfonate is detected within a short period of time in the systemic circulation and tissues without conversion to 2-undecanone.

## 1. Introduction

*H. cordata* is a member of Saururaceae, which is mainly distributed in Asia, including China, Japan, Indian and Korea [1,2,3]. Injections of *H. cordata*, which are normally prepared by volatilization of herb oils, have been used to treat inflammatory-related diseases, such as respiratory infections, conjunctivitis, keratitis, acute and chronic rhinitis and sinusitis [4].

The volatile oil of *H. cordata* contains some olefin compounds, such as pinene, camphene, myrcene and limonene, in addition to decanoyl acetaldehyde (houttuynin, C_12_H_22_O_2_, molecular weight (*M*_W_) 198.3), which is a specific component known to cause “fishy burps”. However, decanoyl acetaldehyde is unstable and is easily converted into 2-undecanone (methyl nonyl ketone, C_11_H_22_O, *M*_W_ 170.3) by steam distillation during the production process. Therefore, 2-undecanone is used as the primary indicator for the quality of *H. cordata* oil. In 1971, sodium houttuyfonate (C_12_H_23_O_5_SNa, *M*_W_ 302.36) was synthesized as a result of the instability of decanoyl acetaldehyde [5], and it has been shown to have a similar pharmaceutical profile to that of decanoyl acetaldehyde, including anti-inflammatory, antibacterial, antiviral and immune enhancing properties [6]. Sodium houttuyfonate has been used clinically for many years to treat upper respiratory tract infections, chronic bronchitis, chronic cervicitis, as well as other conditions. The different available formulations include injection, powder, tablets, suppositories and eye drops.

Some pharmacological studies have shown that sodium houttuyfonate and 2-undecanone have anti-inflammatory efficacy *in vitro* [7,8,9,10]; However, studies are limited, and it is unknown whether sodium houttuyfonate or 2-undecanone exhibits higher activity. Although sodium houttuyfonate is relatively more stable than decanoyl acetaldehyde, it can be converted to decanoyl acetaldehyde and then decanoyl acetic acid via an oxidation reaction. Decanoyl acetic acid can be further transformed to 2-undecanone through a decarboxylation reaction. In a previous study, we prepared sodium houttuyfonate without the presence of 2-undecanone using a steam distillation (SD) method. 2-Undecanone and decanoyl acetaldehyde were identified in the extraction, verifying that sodium houttuyfonate can be converted to 2-undecanone under certain conditions. To the best of our knowledge, this is the first report on the stability of sodium houttuyfonate and will allow for improvements in its clinical applications. This study also addresses whether or not sodium houttuyfonate exerts its anti-inflammatory effects by converting to 2-undecanone *in vivo.*

In our study, RAW264.7 macrophage cells were selected as a model to evaluate the anti-inflammatory activity of sodium houttuyfonate and 2-undecanone *in vitro*. Further studies *in vivo* compared and verified their activities. Specifically, we used a simulated gastrointestinal environment with the primary influencing factors being the solvent, temperature, light, oxidation and pH effects to determine stability. Sodium houttuyfonate was orally administered to mice. The mouse serum and the gastrointestinal tissue were then analyzed by gas chromatography (GC) and gas chromatography-mass spectroscopy (GC-MS) to determine if sodium houttuyfonate can be transformed to 2-undecanone *in vivo*. The goal of this study was to explore whether sodium houttuyfonate or 2-undecanone has higher anti-inflammatory properties and in order to better understand the ideal preparation conditions for maximizing drug efficacy. In addition, these studies provide useful information for avoiding the conversion of decanoyl acetaldehyde in preparations of *H. cordata* volatile oil by SD.

## 2. Results and Discussion

### 2.1. Effects of Drugs on Cell Viability

**Figure 1:**
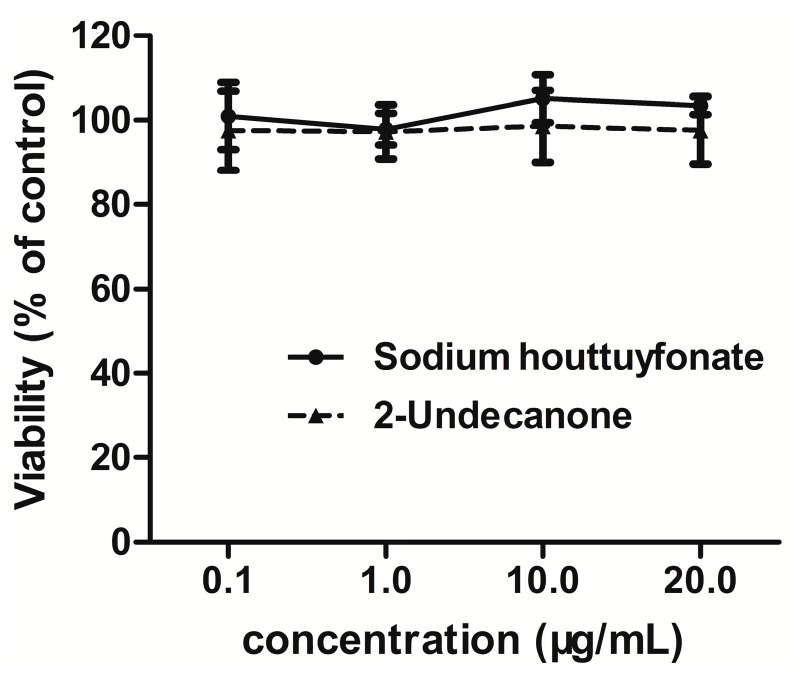
Effects of drugs on cell viability determined by the thiazolyl blue (MTT) assay. Data are expressed as means ± standard deviation (SD).

Treatment of RAW264.7 cells with sodium houttuyfonate and 2-undecanone at concentrations of 0.1–20 μg/mL for 24 h did not cause any cytotoxicity (see Figure 1). In addition, LPS (1 μg/mL) had no effect on the survival of cells (cell viability was 95.6%). However, at higher concentrations (50–100 μg/mL) for 24 h, both sodium houttuyfonate and 2-undecanone induced decreases in the cell survival rate.

### 2.2. Effects of Drugs on Lipopolysaccharide (LPS)-Induced Cytokine Production

**Figure 2:**
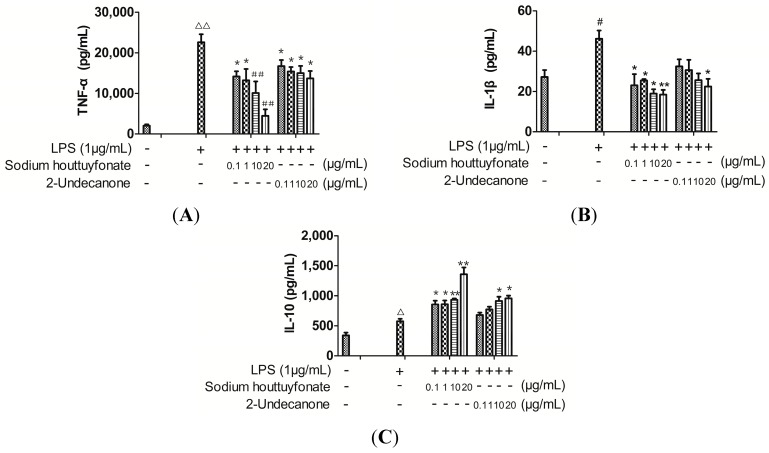
The effects of sodium houttuyfonate and 2-undecanone on cytokine production in lipopolysaccharide (LPS)-induced RAW264.7 cells. (**A**) TNF-α production; (**B**) IL-1β production; and (**C**) IL-10 production in RAW264.7 cells stimulated with LPS and LPS plus sodium houttuyfonate or 2-undecanone. The cells were pre-stimulated with 1 μg/mL of LPS for 1 h, then treated with a series of concentrations (0.1, 1, 10, 20 μg/mL) of sodium houttuyfonate or 2-undecanone and co-incubated for 24 h. The control group was not given drug or LPS, but received an equivalent volume of DMSO. Data are representative of four readings. ^#^ *p* < 0.05, ^Δ^ *p* < 0.01, ^ΔΔ^ *p* < 0.001 *vs.* the control group; ^##^ *p* < 0.001, ** *p* < 0.01, * *p* < 0.05 *vs.* the LPS group.

TNF-α, IL-1β and IL-10 production in the medium of RAW264.7 cells were measured by the enzyme-linked immunosorbent assay (ELISA) (Figure 2). The higher levels of TNF-α and/or IL-1β together with the lower IL-10 levels released by LPS-induced RAW264.7 cells indicate a state of inflammation. Though there is a sodium sulfite ion and a hydroxyl in the structure of sodium houttuyfonate, our experimental data indicate a lack of water solubility. Sodium houttuyfonate can only be suspended in water, and 2-undecanone is also very difficult to dissolve in water. Therefore, dimethyl sulfoxide (DMSO) was used to fully dissolve the two compounds for use in all of the cell experiments. The control group was given an equivalent amount of DMSO. Cytokine levels were calculated according to the standard curve by four-parameter logistic curve fitting. RAW264.7 cells treated with LPS alone resulted in a significant increase in TNF-α, IL-1β and IL-10 production as compared to that of the control group (*p* < 0.001, 0.01 or 0.05). At 0.1–20 μg/mL, sodium houttuyfonate and 2-undecanone exhibited significant decreases in TNF-α and IL-1β levels as compared to the LPS group in a dose-dependent manner (*p* < 0.001, 0.01 or 0.05). There was a dose-dependent increase in the expression of IL-10 after being treated with both sodium houttuyfonate and 2-undecanone (*p* < 0.01 or 0.05). Overall, sodium houttuyfonate had a more obvious influence on cytokine production for TNF-α, IL-1β or IL-10 than 2-undecanone at the same dosage; Demonstrating that sodium houttuyfonate had a stronger anti-inflammatory effect than 2-undecanone at the cellular level (see Figure 2).

### 2.3. Effects of Drugs on LPS-Induced Toll-Like Receptor 4 (TLR4) Expression

TLR4 was the first identified TLR and is essential for the cell response to LPS [11]. To recognize LPS, TLR4 forms a complex with a secreted protein, myeloid differentiation protein-2 (MD-2), which is associated with the extracellular domain of TLR4 [12,13]. LPS binding to MD-2 triggers homodimerization of the TLR4/MD-2 complex and induces the dimerization of TLR4, resulting in the induction of inflammatory signal transduction [14]. TLR4/MD-2 is a key player in inflammation and induces downstream signaling through the formation of an LPS-TLR4/MD-2 complex, which recruits an intracellular adaptor protein, MyD88. MyD88 leads to early activation of mitogen-activated protein kinases (MAPKs) and the transcriptional factor nuclear factor κB (NF-κB) to induce inflammatory cytokine secretion, such as TNF-α [15].

Currently, there is a lack of data on the receptor activity of sodium houttuyfonate and 2-undecanone that would contribute to a further understanding of their respective anti-inflammatory mechanisms. Therefore, in this study, TLR4 expression in LPS-induced RAW264.7 cells was determined after anti-TLR4/MD-2 treatment.

**Figure 3:**
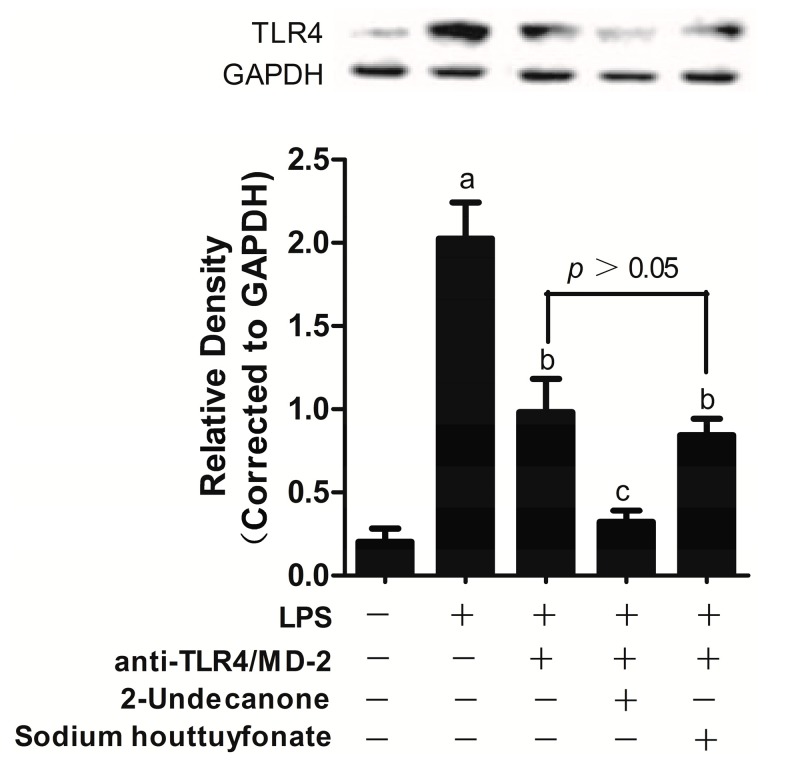
The effects of sodium houttuyfonate and 2-undecanone on the expressions of TLR4 in LPS-induced RAW264.7 cells. **Upper** panel: Western blots for TLR4 protein expression, protein levels of TLR4 were normalized to GAPDH loading control, the picture is of a single representative experiment; **Bottom** panel: TLR4 expression in RAW264.7 cells stimulated with LPS and LPS plus anti-TLR4/MD-2 or the two components. The cells were pretreated with anti-TLR4/MD-2 (10 μg/mL) or plus the two compounds (10 μg/mL) for 1 h prior to stimulation with 1 μg/mL of LPS for 2 h. The control group was not given drugs, LPS or anti-TLR4/MD-2. Data are representative of three independent experiments. ^a^ *p* < 0.001 *vs.* the control group; ^b^ *p* < 0.005 and ^c^ *p* < 0.001 *vs.* the LPS group.

Here, we demonstrate that anti-TLR4/MD-2 treatment inhibited LPS-induced TLR4 expression in RAW264.7 cells compared with the LPS-treated group (*p* < 0.005, Figure 3). Subsequently, treatment with sodium houttuyfonate and 2-undecanone also inhibited LPS-induced TLR4 expression following treatment with anti-TLR4/MD-2 (*p* < 0.005, Figure 3). However, no statistically significant differences were observed between groups with or without sodium houttuyfonate after anti-TLR4/MD-2 treatment, indicating that sodium houttuyfonate may exert its anti-inflammatory effect by blocking the binding of TLR4/MD-2 and LPS. This effect was not observed with 2-undecanone. Consequently, TLR4/MD-2 may be a molecular target for sodium houttuyfonate. This may also explain the difference in anti-inflammatory activity observed between sodium houttuyfonate and 2-undecanone at the same dosage.

### 2.4. Anti-Inflammatory Efficacy in Vivo

**Figure 4:**
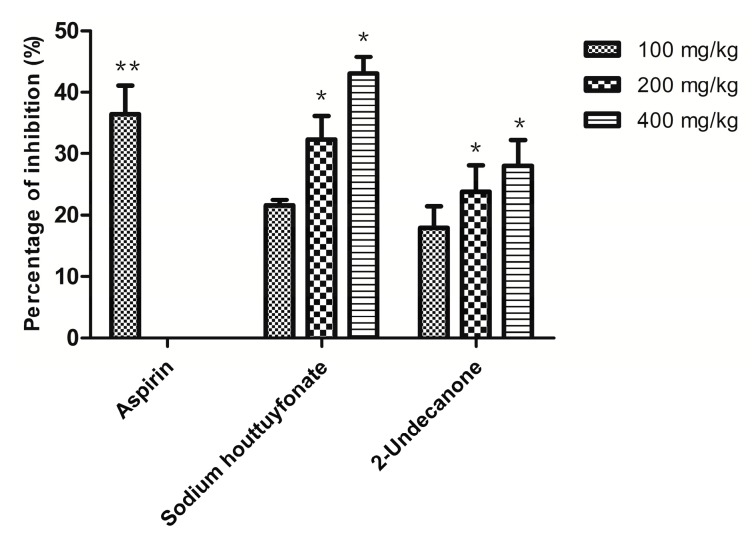
Anti-inflammatory effects of sodium houttuyfonate and 2-undecanone on xylene-induced mouse ear edema. Aspirin is used as the positive control. Data represent the mean of the difference in percentage of inhibition (%) (*n* = 10). ** *p* < 0.01, * *p* < 0.05 *vs.* the model group.

The xylene-induced mouse ear edema method is a typical way to evaluate the topical anti-inflammatory activity of natural products [16]. It has a good predictive value for screening anti-inflammatory agents. He *et al*., [17] found that intravenous (i.v.) injection and oral administration of *H. cordata* and sodium houttuyfonate produced similar metabolites in the serum and urine of rats. In 2006, the China State Food and Drug Administration (CSFDA) temporarily suspended the use of seven *Houttuynia* injective preparations due to adverse drug reactions. However, orally administered sodium houttuyfonate has safety advantages over the injection form; therefore, oral administration was chosen in our study. As a positive control, aspirin (100 mg/kg) significantly inhibited earplug weight by 36.4%. Sodium houttuyfonate showed similar results to aspirin, with a 32.3% reduction in ear swelling at 200 mg/kg (*p* > 0.05). Though 2-undecanone can also attenuate xylene-induced ear edema, it showed lower inhibition than aspirin and sodium houttuyfonate at the same dosage (Figure 4). Treatment with sodium houttuyfonate results in a more significant anti-inflammatory response than 2-undecanone *in vivo* (*p* < 0.05 at 100 mg/kg, *p* < 0.001 at 200 and 400 mg/kg).

Previous reports about the anti-inflammatory mechanism of sodium houttuyfonate have shown an increase in the phosphorylation of calcium/calmodulin-dependent protein kinase-II (CaMK II) and cyclic adenosine monophosphate response element binding protein (CREB). In addition, treatment with sodium houttuyfonate resulted in an increase in the expression of c-Fos protein in macrophages, while the phosphorylation level of extracellular signal-related kinase 1/2 (ERK1/2) was not affected by the treatment [7]. Sodium houttuyfonate has also been shown to protect against cationized bovine serum albumin (C-BSA)-induced glomerulonephritis in BALB/C mice through the suppression of the urine protein, morphological character and monocyte chemotactic protein 1 (MCP-1) (*p* < 0.001) [8]. In addition, sodium houttuyfonate treatment has been shown to induce a respiratory burst and to increase the concentration of free calcium in macrophages, as well as increase IL-2 within T-cells [9]. In contrast to sodium houttuyfonate, experiments *in vitro* revealed that 2-undecanone was able to inhibit LPS-induction of TNF-α, nitric oxide (NO) and H_2_O_2_ production in a dose-dependent manner [10]. Volatile oil of *H. cordata* with 2-undecanone as the main component was found to inhibit the release of LPS-induced prostaglandin E_2_ (PGE_2_) from mouse peritoneal macrophages [18]. Taken together, these studies provide insight into the mechanisms by which treatment with sodium houttuyfonate results in more significant anti-inflammatory activity than 2-undecanone, both *in vitro* and *in vivo*. Specifically, sodium houttuyfonate exerts its anti-inflammatory effect through multiple pathways, including c-Fos, MCP-1 protein and some inflammatory cytokines; However, 2-undecanone exerts its anti-inflammatory effect only by inhibiting some inflammatory mediators.

### 2.5. Studies on Sodium Houttuyfonate Stability (Temperature, Oxidation and Illumination)

#### 2.5.1. Effects of Solvents on Sodium Houttuyfonate Stability

**Figure 5:**
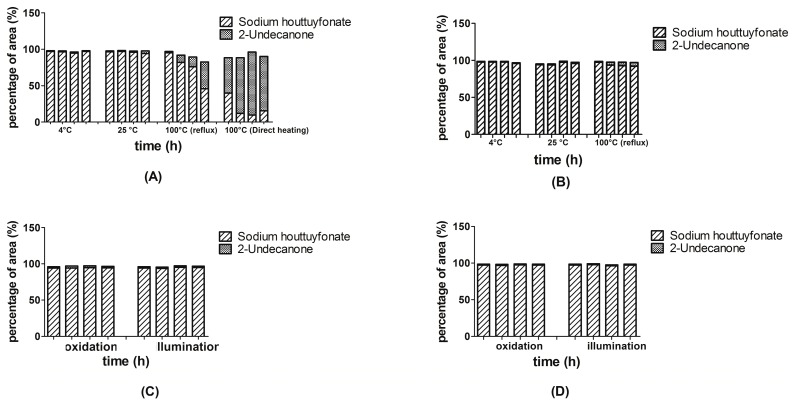
Stability (temperature, oxidation and illumination) of sodium houttuyfonate and 2-undecanone in different solvents. Four columns of each group from left to right were measured for 0.5, 1, 2 and 4 h, respectively. (**A**) temperature (in aqueous solution); (**B**) temperature (in ethyl acetate); (**C**) oxidation and illumination (in aqueous solution); and (**D**) oxidation and illumination (in ethyl acetate).

Previously, we verified that sodium houttuyfonate could be converted to 2-undecanone during SD (in a water bath at 100 °C). Therefore, in this experiment, we focused primarily on the properties of solvency and temperature. We chose 4, 25 and 100 °C (the boiling point of aqueous solution) as the experimental temperatures. Figure 5 shows that solvent and temperature influence the stability of sodium houttuyfonate. Sodium houttuyfonate remained stable both in an aqueous solution and ethyl acetate after refrigeration for 4 h. However, at 25 °C for 4 h, sodium houttuyfonate slightly degraded to form 2-undecanone in an aqueous solution, but remained stable in ethyl acetate. In contrast, sodium houttuyfonate converted quickly in aqueous solution when heated at reflux, but was relatively stable in ethyl acetate (only slightly transformed). When directly heated in an aqueous solution, sodium houttuyfonate degraded to 2-undecanone almost entirely within 1 h.

Sodium houttuyfonate was stable under oxidation and illumination both in aqueous solution (relative standard deviation (RSD) = 0.23% and 0.97%, respectively) and ethyl acetate (RSD = 0.35% and 0.68%, respectively) as seen from the percentage of peak areas. Under conditions of oxidation and illumination at 4 °C, sodium houttuyfonate was not converted to 2-undecanone in either aqueous solution or ethyl acetate.

Thus, the key factors in the stability of sodium houttuyfonate and 2-undecanone were the solvent followed by temperature. In particular, an aqueous solution and higher temperatures have a pronounced effect on the stability of sodium houttuyfonate.

#### 2.5.2. Sodium Houttuyfonate Stability in the Aqueous Solutions at Different pH Values

**Figure 6:**
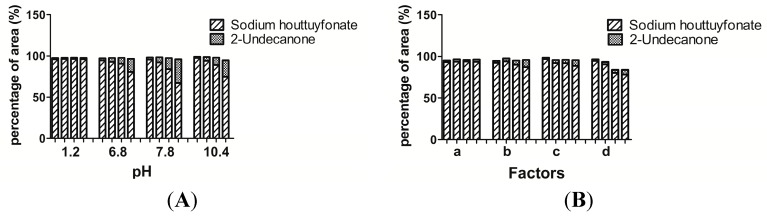
Stability of sodium houttuyfonate under different conditions. Four columns of each group from left to right were measured at 0.5, 1, 2 and 4 h, respectively. (**A**) Effects of pH; and (**B**) simulated gastrointestinal environments. a: Simulated gastric fluid; b: Solution of pepsin; c: Simulated intestinal fluid; and d: Solution of trypsin. The experiments were conducted in a water bath at 37 °C.

Sodium houttuyfonate was stable (RSD 0.14%) in a solution of pH 1.2, but degraded into 2-undecanone to various degrees at a pH range of 6.8 to 10.4, as seen from the percentage of peak area (Figure 6). In a solution of pH 7.8, the maximum degradation was 67.33% in 4 h. In order to be consistent with the temperature of simulated gastric and intestinal solutions *in vitro*, the experiments were conducted in a water bath at 37 °C. Sodium houttuyfonate was unstable in neutral and alkaline solutions. It should be further verified if sodium houttuyfonate degrades into 2-undecanone in an intestinal solution after oral administration.

Although sodium houttuyfonate was stable in a solution of pH 1.2 (simulated gastric solution without pepsin), we subsequently examined the impact of pepsin on its stability.

#### 2.5.3. Stability Study of Sodium Houttuyfonate under the Influence of Simulated Gastric and Intestinal Conditions

In order to explore the stability and possible metabolism of sodium houttuyfonate *in vivo*, standard simulated gastric and intestinal environments were created *in vitro* and their effects were studied. Similar to the results seen in a solution of pH 1.2 (without pepsin), sodium houttuyfonate was also stable in simulated gastric fluid (pH = 1.3) containing pepsin and only slightly degraded into 2-undecanone at 4 h. In a solution of pepsin (pH = 3.6), sodium houttuyfonate degraded to 87.24% at 4 h. This effect is thought to be a consequence of the change in pH rather than due to the presence of pepsin.

In contrast to a simulated gastric environment, sodium houttuyfonate was unstable in both a simulated intestinal fluid (pH = 6.8, with trypsin) and a solution of trypsin (pH = 3.3) with a degradation of 11.26% and 21.74% in 4 h, respectively (Figure 6). Sodium houttuyfonate was less degraded in a simulated intestinal fluid (pH = 6.8) containing trypsin than in a solution without trypsin, degrading to 80.65% in 4 h (Figure 6). In contrast, sodium houttuyfonate degraded to 67.33% within 4 h in a simulated colonic fluid (pH = 7.8) without trypsin. However, this may be due to the package effect of trypsin (trypsin was separated from water into white matter, which adhered to the wall of the flask), resulting in its relative stability at a similar pH.

### 2.6. Preliminary Study on Bioavailability of Sodium Houttuyfonate

In order to confirm the *in vitro* stability results for sodium houttuyfonate in a simulated gastrointestinal environment, we conducted further testing using an *in vivo* mouse model*.* The blood and gastrointestinal tissues of mice were harvested at different times after oral administration of sodium houttuyfonate; identification and quantification of sodium houttuyfonate were determined using GC and GC-MS techniques. In this study, sample extraction with ethyl acetate failed to detect either sodium houttuyfonate or 2-undecanone in serum and tissue samples. These results were consistent with other published data using *n*-hexane [17]. In order to be detected by GC or high performance liquid chromatography (HPLC), sodium houttuyfonate must first be transformed into decanoyl acetaldehyde by a hydrolysis reaction in alkali conditions. Although sodium houttuyfonate can be detected by HPLC in the alkaline mobile phase (containing 0.1 M NaOH) [19], 2-undecanone cannot be simultaneously detected by HPLC, but can be detected by GC. In order to detect sodium houttuyfonate and 2-undecanone by GC and GC-MS at the same time, a method of sample preparation was utilized that required the addition of a sodium carbonate solution (0.01 M) to the serum and tissue samples followed by ethyl acetate extraction. Obvious chromatographic peaks were observed at the retention time that corresponds with decanoyl acetaldehyde, the degradation product of sodium houttuyfonate.

**Figure 7:**
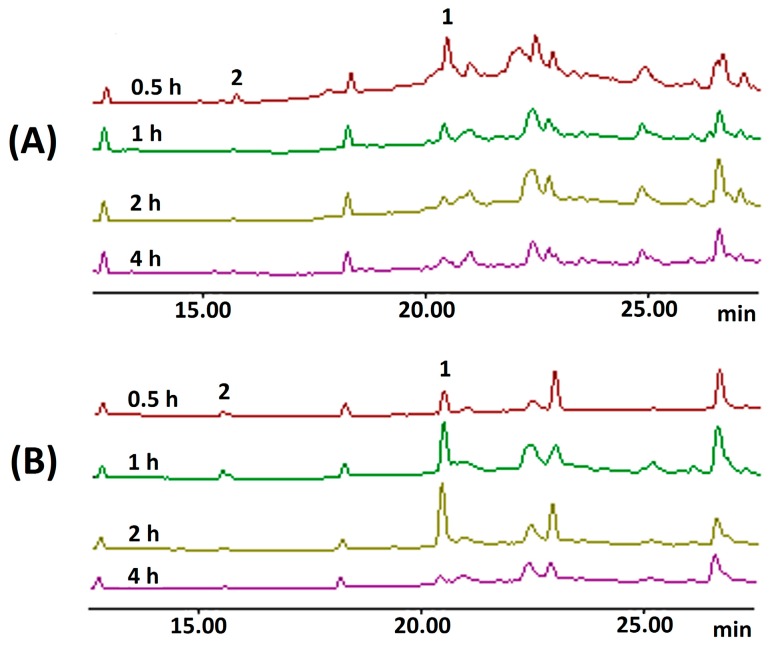

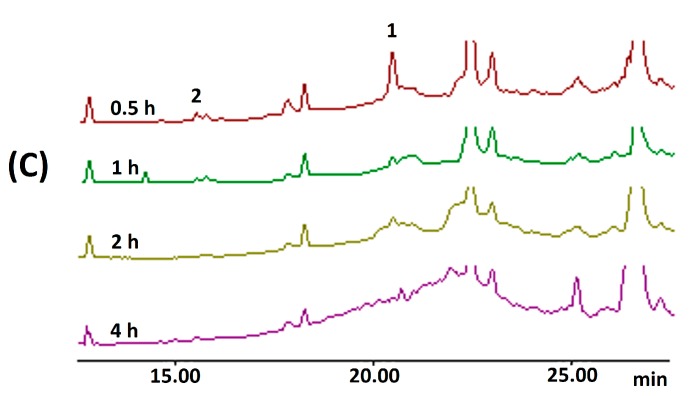
GC chromatography of samples *in vivo*. (**A**) Serum samples, *y*-axis: 0.7 × 10^5^; (**B**) samples of stomach, *y*-axis: 1.0 × 10^5^; and (**C**) samples of intestine, *y*-axis: 0.3 × 10^5^. 1, sodium houttuyfonate; 2, 2-undecanone.

To ensure that the observed results were reliable, a comparison was made of the relative peak areas associated with the entry of sodium houttuyfonate into the body. Sodium houttuyfonate in the serum, stomach and intestine can be detected immediately by GC at 0.5 h. The concentration of sodium houttuyfonate in the stomach gradually increased from 0.5–2 h, but was drastically reduced at 4 h. Furthermore, the concentration in the serum and intestine at 1–4 h decreased in a time-dependent manner, indicating that sodium houttuyfonate can be quickly absorbed into the circulatory system and intestine and might be quickly distributed to other organs, such as trachea, lung, brain, heart and kidney, although this requires further verification (See Figure 7).

**Figure 8:**
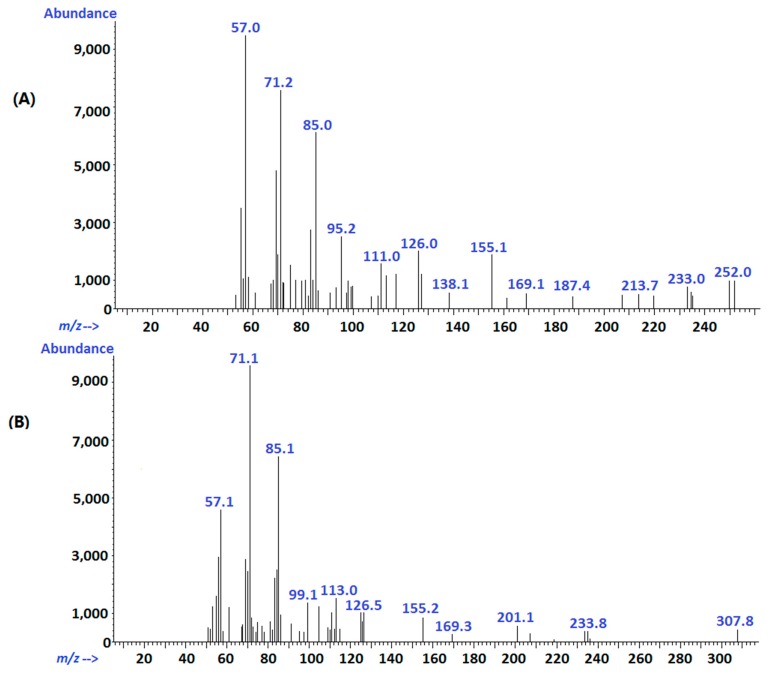
MS fragment ions of 2-undecanone (**A**) and decanoyl acetaldehyde (**B**).

The active aldehyde α-H in decanoyl acetaldehyde can easily lead to the degradation to 2-undecanone through a condensation reaction under alkaline conditions [8]. Therefore, we also detected chromatographic peaks of 2-undecanone in the sample solutions at 0.5 h. Since it was not detected either in serum or tissue samples without alkali treatment, the 2-undecanone detected at 0.5 h was not a degradation product from sodium houttuyfonate *in vivo*, but generated during sample processing. Qualitative analysis of serum and tissue samples by GC-MS showed that the molecular ion peaks at the retention time of 17.4 and 23.1 min were 170 and 198, respectively. That is consistent with the molecular ions of 2-undecanone and decanoyl acetaldehyde; However, the compounds, 2,3-dimethyldecane (*M*_W_ 170) and tridecane, 6-methyl (*M*_W_ 198), are present in the spectrum, possibly in low concentrations *in vivo*, and some MS fragments were not consistent with the NIST Mass Database (2008). Nonetheless, the main fragments were consistent with their pyrolysis patterns, and further analysis and comparison with the GC chromatography of standard solutions confirmed the identity of 2-undecanone and decanoyl acetaldehyde. Figure 8 shows the MS fragment peaks of the two substances. For 2-undecanone (*M*_W_, 1169), the major fragment ions were *m*/*z* 71[M-CH_3_(CH_2_)_3_CO]^+^ and *m*/*z* 57[CH_3_COCH_2_]^+^; the other fragment ions belonged to *m*/*z* 155[M-CH_3_]^+^ and *m*/*z* 126[M-CH_3_COH]^+^. The molecular ion peak for decanoyl acetaldehyde was not observed, possibly due to the instability of its molecular ion; however, the major fragment ions were *m*/*z* 85[CHOCH_2_COCH_2_]^+^, 71[CHOCH_2_CO]^+^ and 57[CH_3_(CH_2_)_3_]^+^.

The *in vivo* results confirm that sodium houttuyfonate is not transformed into 2-undecanone after it enters the body, which is consistent with the results of the simulated gastrointestinal experiments *in vitro*.

## 3. Experimental Section

### 3.1. Reagents and Materials

2-Undecanone (content 99%) was purchased from Sigma-Aldrich (Shanghai) Trading Co., Ltd., (Shanghai, China). Sodium houttuyfonate (content 99%) was from Shanghai Qing-ping Pharmaceutical Co., Ltd., (Shanghai, China). Ethyl acetate (analytical grade) was obtained from Tianjin (Hong Kong) Xin-tong Fine Chemical Co., Ltd., (Tianjin, China). Other reagents were of analytical grade.

RAW264.7 cells were obtained from the Center Laboratory of Molecular Biology in Tongji Hospital of Tongji medical college, Huazhong University of Science and Technology (Wuhan, China). Kits of TNF-α, IL-1β and IL-10 were obtained from eBioscience, Inc., (San Diego, CA, USA). Roswell Park Memorial Institute (RPMI) 1640 medium was purchased from KeyGEN Bio TECH (Nanjing, China). LPS was purchased from Sigma-Aldrich Biotechnology (St. Louis, MO, USA). Fetal bovine serum (FBS) was obtained from HyClone (Logan, UT, USA). Tween-20, DMSO and 1-(4,5-dimethylthiazol-2-yl)-3,5-diphenylformazan (MTT) were from Promoter Bio Com. (Wuhan, China). Trypsin-ethylene diamine tetraacetic acid (EDTA) digestive juice was purchased from Gino Biomedical Technology Co., Ltd., (Hangzhou, China). Anti-TLR4/MD-2 was obtained from eBioscience, Inc. Rabbit anti-mouse anti-TLR4 and anti-glyceraldehyde-3-phosphate dehydrogenase (GAPDH) were obtained from Cell Signaling Technology (CST, Boston, MA, USA).

### 3.2. Animals

Male KM mice in clean grade (18–20 g body weight) were provided by the Laboratory Animal Center of Tongji medical college, Huazhong University of Science and Technology (Wuhan, China). Mice were housed in controlled environmental conditions (temperature, 25 ± 2 °C; relative humidity, 70% ± 5%), with free access to standard food and water. The experimental animals were fasted for 12 h with free access to water prior to the experiments. All experiments were conducted in accordance with the animal protocols and guidelines approved by the Medicine Ethics Review Committee for animal experiments, which is in compliance with the internationally accepted principles for laboratory animal use and care, as found in the European Community guidelines (EEC Directive of 1986; 86/609/EEC).

### 3.3. Apparatus and Chromatographic Conditions

An Agilent 6890N series GC system (Agilent Technologies Co., Ltd., Palo Alto, CA, USA) equipped with a hydrogen flame ionization detector was applied for quantitative analysis. Separation was achieved with an Agilent HP-5 capillary column (30 m × 0.32 mm, 0.25 μm, Agilent Technologies. The carrier gas was nitrogen with flow of 1.2 mL/min. The oven temperature was programmed at 60 °C for 5 min, with a rise of 5 °C/min up to 210 °C and held for 5 min. The temperature of the injector and detector were set at 250 and 300 °C, respectively. The splitless injection volume was 1 μL.

The determination of components in the serum and tissue samples was carried out by GC-MS (Agilent Technologies). The GC-MS system was comprised of an Agilent 6890N series GC connected to an Agilent 5925B mass selective detector (negative ion mode). An Agilent HP-5 capillary column (30 m × 0.25 mm, 0.25 μm, Agilent Technologies) was used with other conditions as described above. All mass spectra were recorded in the full scan mode at 70 eV (*m*/*z* 50–500) and all data acquisition was performed by CLASS-5000 Chemstation Software (Agilent Technologies).

An enzyme mark instrument (Thermo Scientific, Palo Alto, CA, USA) was used for cytokine detection. A CO_2_ incubator (Thermo Scientific), an AUW220D electronic balance (SHIMADZU, Kyoto, Japan), an illumination instrument and an electric ear-swelling puncher were used in trials.

### 3.4. Preparation of Solutions

A stock solution of 2-undecanone was prepared in ethyl acetate at a concentration of 2.76 mg/mL and stored at 4 °C before use. A solution of sodium houttuyfonate was prepared just before use according to the reference method [20] at a concentration of 4.5 mg/mL.

### 3.5. Anti-Inflammatory Efficacy in Vitro

LPS-induced inflammation of Raw264.7 cells was used as a model for measuring the anti-inflammatory efficacy of sodium houttuyfonate and 2-undecanone *in vitro*.

#### 3.5.1. Cell Viability Assay

Cell viability following drug exposure was determined by the mitochondrial-dependent reduction of MTT to formazan crystals [21]. Specifically, cells were distributed into each well of a 96-well plate (10^5^ cells/well) (Corning, Wilmington, NC, USA) and incubated overnight. The cells were then treated with six different concentrations of sodium houttuyfonate or 2-undecanone (final 0.1–100 μg/mL) and LPS (final 1.0 μg/mL) for 24 h and then exposed to 10 μL of MTT (5 mg/mL) and incubated at 37 °C for 4 h. The solubilization solution (100 μL) included in the MTT kit was then added to each well. After overnight incubation, we used an enzyme mark instrument to measure the optical densities of the 96-well culture plates at 570 nm. The optical densities of untreated control cells were taken as 100%.

#### 3.5.2. Determination of Cytokine in RAW264.7 Cells

Methods of cell culture and drug stimulation were as previously described with a final volume of 240 μL. After 24 h of incubation at 37 °C, 100 μL of supernatants diluted with 10- or 20-fold phosphate buffer solution (PBS) were collected for the cytokine assay. We then measured levels of cytokines using a TNF-α, IL-1β or IL-10 ELISA kit.

#### 3.5.3. Western Blot Analysis on TLR4 Expression

RAW264.7 cells were cultured in RPMI 1640 medium, containing 10% FBS. Cells were distributed into each well of a 6-well plate (5 × 10^5^ cells/well) (Corning). After overnight incubation, cells were synchronization-treated in RPMI 1640 medium containing 0.5% FBS for 24 h. Then, the cells were pretreated with anti-TLR4/MD-2 (10 μg/mL) plus sodium houttuyfonate or 2-undecanone (10 μg/mL) for 1 h prior to stimulation with LPS (1 μg/mL) for 2 h. Samples lacking treatment of drugs, LPS and anti-TLR4/MD-2 were designated controls.

Following the addition of LPS, cells were washed three times with cold PBS, and the residual PBS in each well was removed after being kept at 4 °C for 0.5 h. The cells were then lysed in iced RIPA lysis buffer for 30 min. Lysates were obtained immediately after centrifugation at 12,000 rpm for 15 min at 4 °C and then subjected to dodecyl sulfate, sodium salt (SDS)-Polyacrylamide gel electrophoresis (PAGE) and Western blot analysis. TLR4 protein was detected with anti-TLR4 polyclonal antibody.

The data are presented as the mean ± standard deviation (SD). Statistical significance was evaluated using a Student’s *t*-test.

### 3.6. Anti-Inflammatory Efficacy in Vivo

The xylene-induced ear edema test was performed in order to evaluate the anti-inflammatory activity of the two compounds orally administrated at 100, 200 or 400 mg per 1 kg bodyweight in mice. Mice were randomly divided into eight groups (*n* = 10). Control animals received an equal volume of 2% Tween-80 (sample solvent), while aspirin (100 mg/kg) served as the reference. A total of 20 μL of xylene was applied to the inner and outer surface of the right ear of each mouse 60 min after the last application of the treatment. The left ear remained untreated. The mice were sacrificed by cervical dislocation 30 min later, and the plugs (8 mm in diameter) were removed with an electric ear-swelling puncher from both ears. The difference in weight between the two plugs was taken as a measure of edematous response.

### 3.7. Studies on Sodium Houttuyfonate Stability (Temperature, Oxidation and Illumination)

#### 3.7.1. Effects of Solvents on Sodium Houttuyfonate Stability

Sodium houttuyfonate (about 90.0 mg) was added to 50 mL of sodium carbonate solution (0.01 M) with shaking extraction in an ice water bath for 15 min. Appropriate amounts of solution were pipetted and separately placed in one of five conditions as follows: (1) Under refrigeration at 4 °C with the flask opened or closed; (2) At a constant temperature of 25 °C; (3) In a water bath heated to reflux (100 °C); (4) Under direct heating at 100 °C; or (5) Within an illumination instrument, including an ice bath, closed flask and light intensity of 4500 Lx ± 500 Lx. Subsequently, 5 mL of solution was extracted with 5 mL of ethyl acetate at 0.5, 1, 2 or 4 h, respectively. Anhydrous sodium sulfate was then used to dry the samples. The process was then repeated with the addition of sodium houttuyfonate (about 90.0 mg) to 50 mL of sodium carbonate solution (0.01 M) and 50 mL of ethyl acetate. All prepared solutions were analyzed under the chromatography conditions described above.

#### 3.7.2. Sodium Houttuyfonate Stability in the Aqueous Solutions at Different pH Values

Aqueous solutions at different pH values were prepared according to the methods from the Chinese Pharmacopoeia [22]. Simulated gastric fluid without pepsin was prepared as follows: 9 mL of concentrated hydrochloric acid and 800 mL of water were mixed well, then diluted to 1000 mL (pH 1.2). Similarly, simulated intestinal fluid without trypsin was prepared as follows: 6.8 g of KH_2_PO_4_ was dissolved in 500 mL of water, then diluted to 1000 mL with pH adjusted to 6.8 by NaOH. The simulated colon fluid was prepared by adding 5.59 g of K_2_HPO_4_ and 0.41 g of KH_2_PO_4_ to the appropriate water until dissolved, then diluted to 1000 mL with pH adjusted to 7.8. Proper amounts of sodium houttuyfonate (about 90.0 mg) were added to 50 mL of the aqueous solutions prepared above or sodium carbonate solution (pH 10.4, 0.01 M), then were shook in an ice water bath for 15 min and placed in a water bath at 37 °C. Solutions were drawn out in 0.5, 1, 2, 4 h and extracted with ethyl acetate. After drying with anhydrous sodium sulfate, the sample solutions were analyzed.

#### 3.7.3. Stability Study of Sodium Houttuyfonate under the Influence of Simulated Gastric and Intestinal Conditions

Simulated gastrintestinal fluids were prepared according to the methods from the Chinese Pharmacopoeia [22]. The solution that only included pepsin or trypsin was 1000 mL of double-distilled water with the addition of 10 g of pepsin or trypsin, respectively.

### 3.8. Preliminary Study on Bioavailability of Sodium Houttuyfonate

Sodium houttuyfonate was orally administrated at 100 mg/kg to mice. Blood samples were drawn from mice into heparinized Epoxy epoxide (EP) tubes at 0.5, 1, 2 and 4 h. Simultaneously, organs, including the stomach and bowel, were immediately removed, washed in normal saline, blotted dry with filter paper and stored at −20 °C until analysis. Blood samples were immediately procured by centrifugation at 3000 rpm for 10 min, then serum was transferred into a new 1.5 mL EP tube and stored at −20 °C until analysis. Serum samples (500 μL) were extracted by ethyl acetate (200 μL) with or without a sodium carbonate solution (0.01 M, 500 μL) added before extraction. After centrifugation at 3000 rpm for 10 min, the supernatants from the ethyl acetate extracts were analyzed by GC and GC-MS. Samples of sodium houttuyfonate and 2-undecanone with a blank serum prepared as described above were analyzed simultaneously. Accurately weighed amounts of the tissues (0.1–0.4 g) were individually cut up and then extracted by ethyl acetate (2 mL) with or without a sodium carbonate solution (0.01 M, 5 mL) added before extraction. GC and GC-MS methods were used to analyze the sample solutions of tissues simultaneously.

## 4. Conclusions

Sodium houttuyfonate and 2-undecanone are two important components from *H. cordata* essential oil that show promise for clinical applications. However, there is a lack of comparative studies on the anti-inflammatory efficacy between sodium houttuyfonate and 2-undecanone. Furthermore, there is a lack of data regarding the potential for sodium houttuyfonate to be converted to 2-undecanone during the SD process. Therefore, these studies compared the anti-inflammatory activity of sodium houttuyfonate and 2-undecanone, as well as the overall stability of sodium houttuyfonate. Results of the *in vitro* cell experiments, as well as the *in vivo* anti-inflammatory experiments showed that sodium houttuyfonate had better anti-inflammatory bioactivity than 2-undecanone at the same dosage. This phenomenon is partially explained by our experiment, as well as others, on TLR4 expression in the LPS-TLR4/MD-2 pathway. Additionally, the inhibitory activity of *H. cordata* essential oil elicited a dose-dependent inhibition of COX-2 enzyme activity (IC_50_ value: 30.9 μg/mL) from mouse peritoneal macrophages, suggesting a role for the arachidonic acid metabolism pathways. *H. cordata* was also found to cause a reduction in LPS-induced COX-2 mRNA and protein expression, but did not affect COX-1 expression [18]. Future studies will include measuring the effect of sodium houttuyfonate or 2-undecanone on COX-2 expression.

Studies on the stability and preliminary bioavailability of sodium houttuyfonate *in vitro* and *in vivo* demonstrated that the main influencing factors are solvent, temperature and pH value. Sodium houttuyfonate is relatively stable in ethyl acetate and in solutions with pH values lower than 1.2, but its stability decreased in solutions with increasing pH values. High temperature can accelerate the degradation of sodium houttuyfonate in these circumstances. Sodium houttuyfonate is slightly affected by pepsin and trypsin, but not by oxidation and illumination. *In vivo*, sodium houttuyfonate can enter the blood circulation without conversion to 2-undecanone and might quickly enter other tissues, except the stomach and intestine, after oral administration. Though under certain conditions *in vitro*, sodium houttuyfonate is degraded into 2-undecanone, its primary anti-inflammatory activity *in vivo* is due to the parent compound. The above studies will benefit the producible quality and stability of sodium houttuyfonate formulations and their clinical application.
